# Achaete-Scute Homolog 1 Expression Controls Cellular Differentiation of Neuroblastoma

**DOI:** 10.3389/fnmol.2016.00156

**Published:** 2016-12-21

**Authors:** Mumtaz Kasim, Vicky Heß, Holger Scholz, Pontus B. Persson, Michael Fähling

**Affiliations:** Institut für Vegetative Physiologie, Charité-Universitätsmedizin BerlinBerlin, Germany

**Keywords:** differentiation therapy, retinoic acid, ASCL1, hASH1, hypoxia, MYCN

## Abstract

Neuroblastoma, the major cause of infant cancer deaths, results from fast proliferation of undifferentiated neuroblasts. Treatment of high-risk neuroblastoma includes differentiation with retinoic acid (RA); however, the resistance of many of these tumors to RA-induced differentiation poses a considerable challenge. Human achaete-scute homolog 1 (hASH1) is a proneural basic helix-loop-helix transcription factor essential for neurogenesis and is often upregulated in neuroblastoma. Here, we identified a novel function for hASH1 in regulating the differentiation phenotype of neuroblastoma cells. Global analysis of 986 human neuroblastoma datasets revealed a negative correlation between hASH1 and neuron differentiation that was independent of the N-myc (MYCN) oncogene. Using RA to induce neuron differentiation in two neuroblastoma cell lines displaying high and low levels of hASH1 expression, we confirmed the link between hASH1 expression and the differentiation defective phenotype, which was reversed by silencing hASH1 or by hypoxic preconditioning. We further show that hASH1 suppresses neuronal differentiation by inhibiting transcription at the RA receptor element. Collectively, our data indicate hASH1 to be key for understanding neuroblastoma resistance to differentiation therapy and pave the way for hASH1-targeted therapies for augmenting the response of neuroblastoma to differentiation therapy.

## Introduction

Neuroblastoma, a malignant tumor derived from the sympathetic nervous system, represents one of the most common solid childhood tumors. They are classified into different stages based on genetic profile, age of onset, and tumor stage, with amplification of the N-myc (MYCN) gene seen in 22% of primary tumors and associated with worse clinical outcome (Cohn et al., 2009; Brodeur and Bagatell, 2014). Gene expression profiles suggest neuroblastomas to be locked in development at an early stage, being irresponsive to the normal cues that trigger differentiation. They, however, have the capacity to differentiate into mature cells in response to a variety of physiological and pharmacological agents. Thus, retinoic acid (RA), by its ability to induce differentiation, remains the first line of therapy for high-risk neuroblastoma (Reynolds et al., 2003). The greatest obstacle to differentiation therapy lies in their refractiveness to RA and it is therefore desirable to understand the mechanisms of resistance and to identify means by which to improve RA effectiveness.

The transcription factor achaete-scute homolog 1 protein (hASH1 for human and Mash1 for mammalian), encoded by the achaete-scute complex-like 1 (*ASCL1*) gene and hereafter referred to as hASH1, is expressed in neural crest cells and neural crest derived progenitor cells of the sympathoadrenal system and is required for neurogenesis (Castro et al., 2011; Imayoshi et al., 2013; Jacob et al., 2013). The hASH1 transcriptional program and regulation of its activity have been well documented (Castro et al., 2011; Imayoshi et al., 2013; Huang et al., 2014; Raposo et al., 2015; Wylie et al., 2015). Detailed characterization of the transcription factor activity has previously revealed that it activates target genes involved in both proliferation and differentiation that are associated with its oscillatory or sustained mode of expression, respectively (Castro et al., 2011; Imayoshi et al., 2013). During development, hASH1 expression is mostly restricted to the embryonic state, being regulated spatially and temporally primarily by transcriptional inhibition by the Notch pathway (Axelson, 2004; Huang et al., 2014). hASH1 is also regulated by post-transcriptional mechanisms involving both mRNA stability and translation (Fähling et al., 2009; Benko et al., 2011). One such important post-transcriptional regulator of hASH1 expression is the heterogeneous nuclear ribonucleoprotein A2/B1 (hnRNP A2/B1) and its role has been described in neuroblastoma cells exposed to hypoxia, indicating that low oxygen tension could be an important determinant of neuroendocrine development and tumor development (Kasim et al., 2014). Surprisingly, aberrantly high levels of hASH1 have been detected only in neuroblastoma cell lines; these being mostly derived from highly malignant tumors and high hASH1 levels thus associated with poor clinical outcome (Isogai et al., 2011). Gaining insight into the regulatory cues leading to hASH1 down-regulation during development could therefore be important for understanding neuroblastoma. Consistently, expression of hASH1 is down-regulated during differentiation, independent of differentiation agent. Whether the down-regulation of hASH1 is essential for differentiation or a consequence thereof could not be clearly established as stable cell clones of the SH-SY5Y cell line expressing high levels of hASH1 were not tolerated (Söderholm et al., 1999). Thus, a functional link between hASH1 expression and the responsiveness of neuroblastoma to differentiation remains elusive (Söderholm et al., 1999; Grynfeld et al., 2000).

Here, we exploit two neuroblastoma cell lines, Kelly and SH-SY5Y as paradigms of malignant tumors expressing endogenously high and low levels of hASH1, respectively, to investigate the functional importance of hASH1 in neuroblastoma. By analysis of three publicly available microarray datasets of human neuroblastoma patients (*n* = 986) and by experiments performed in the above mentioned cell lines, we identify a crucial role for hASH1 in regulating the differentiation potential of neuronal cells via its ability to repress RA-mediated transcription. These results not only expand the repertoire of hASH1 functions but also help to explain and eventually overcome the refractiveness of many neuroblastomas to differentiation therapy. Our data further reveal the functionality of hASH1-targeted therapies for augmenting the response of neuroblastoma to differentiation therapy.

## Materials and Methods

### Microarray Data Analysis

We used the R2: Genomics Analysis and Visualization web tool to find genes correlated with hASH1 (ASCL1). We included the following three public datasets for analysis: the Versteeg dataset (GSE16476) which included 88 human neuroblastoma samples (Valentijn et al., 2012), the Asgharzadeh dataset (Therapeutically Applicable Research to Generate Effective Treatments initiative^1^) included 249 human neuroblastoma samples (Russell et al., 2015) and the Kocak dataset (GSE45547) with 649 human neuroblastoma samples (Kocak et al., 2013), all with different clinical characteristics. We applied a *p*-value cut-off of 0.01 for the correlation coefficient (*R* value) to obtain significantly correlated genes. The *p* values were corrected for multiple testing according to the false discovery rate. Only for the Kocak dataset that yielded >4500 correlated genes, we applied an additional *R*-value cutoff of ≤ −0.3 or ≥ +0.3. Gene ontology (GO) analysis (database release date 2016-04-23), using the Bonferroni correction for multiple testing and a cutoff *p* value < 0.05, was independently performed on the list of significantly correlated genes obtained from each of the three datasets.

### Cell Culture and Treatments

The human neuroblastoma cell lines, Kelly (ACC 355) and SH-SY5Y (CRL-2266), were grown at 37°C, 5% CO_2_ in RPMI medium supplemented with 10% Fetal Bovine Serum (FBS). Cells were differentiated with 1 μM and 10 μM all-trans RA for the indicated times. For shorter treatment times (up to 24 h), cells that had outgrowths greater than the length of the cell body were considered to be expressing neurites. For longer treatment times (4 days), cells that had one or more outgrowths that reached at least double the diameter of the cell body were considered to be differentiated. For hypoxia, a hypoxic chamber set at 1% O_2_, 37°C and 5% CO_2_ was used.

### Antibodies

The following antibodies were used: mouse monoclonal anti-Mash1 (murine homolog of hASH1; BD Pharmingen), mouse monoclonal anti-hnRNP-A2/B1 (Acris Antibodies, Rockville, MD, USA), rabbit polyclonal anti-tubulin (Proteintech), rabbit polyclonal anti-neurofilament L (Proteintech). Secondary antibodies used were donkey anti-rabbit and goat anti-mouse IgG-HRP (Santa Cruz Biotechnology, Santa Cruz, CA, USA). The Cy3-coupled secondary antibody for immunofluorescence was from Jackson ImmunoResearch Laboratories.

### Protein Isolation and Western Blot

Total cellular extracts were prepared by direct lysis of cells in 50 mM Tris (pH 6.8) buffer containing 4 M Urea and 1% SDS. Western blotting was performed as previously described (Fähling et al., 2006).

### Real Time PCR Analysis

Total RNA was prepared using RNA-Bee (Biozol Diagnostica Vertrieb GmbH) and real-time PCR experiments performed as previously described (Kasim et al., 2014). Each sample was measured in triplicate. mRNA expression levels were normalized to 18S rRNA using the ΔΔC_t_ method. Primer sequences used are listed in Supplementary Table S1.

### RNA Interference

Control siRNA and siRNA targeting hASH1 and hnRNP-A2/B1 was transfected into Kelly cells using SilenceMag (Oz Biosciences) or DharmaFECT 2 (Thermo Scientific, Waltham, MA, USA) as specified by the manufacturer. All siRNAs were purchased as SMARTpool siRNAs from Thermo Scientific Dharmacon. Cells were analyzed 48 h post-transfection for knock-down efficiency or used for further experiments at 24 h or 48 h post-transfection.

### Cell Transfection and Luciferase Reporter Assays

Control and backbone vectors were purchased directly from Clontech and Promega. For knock-down and overexpression experiments, cells were grown in 24-well plates. Following 24 h knockdown in Kelly cells, a control luciferase (CONTROL-LUC) plasmid or the RA receptor element containing luciferase plasmid along with the renilla luciferase phRL-TK vector (Promega) was co-transfected using PolyMag (Oz Biosciences). For over-expression of hASH1 in SH-SY5Y, a pCMV-Sport6-hASH1 plasmid or control plasmid was co-transfected with the reporter constructs. After an overnight incubation, the medium was changed and RA was supplemented at a final concentration of 1 μM. Cells were grown for an additional 18 h. Luciferase activity was measured using the Dual-Glo^TM^ luciferase assay system and the data collected using a luminometer (Berthold Detection Systems). Co-transfection of hASH1 with EGFP in SH-SY5Y was performed in 6-well plates with a final concentration of 2 μg DNA. The ratio of hASH1 to EGFP plasmid was maintained at 9:1.

### Microscopy

The cell nuclei were counterstained with 4′,6-diamidino-2-phenylindole (DAPI). Immunofluorescence was performed as recommended in the antibody data sheet. An epifluorescence microscope (Axiovert100, Carl Zeiss, Berlin, Germany) connected to a digital camera (SPOT RT Slider, Diagnostic Instruments, Sterling Heights, MI, USA) with SPOT software (Universal Imaging Corp., Marlow Buckinghamshire, UK) was used for image acquisition. Where indicated, image analysis was performed using the ImageJ software with the NeuronJ plugin.

### Statistics

If not indicated otherwise, all values are presented as mean ± SD of at least three independent experiments, each with three biological replicates. Student’s paired *t* test was applied to reveal statistical significances between two samples. To compare means of three or more groups, a one-way ANOVA was used. *p* < 0.05 was considered significant.

## Results

### hASH1 Is Negatively Correlated with Genes Involved in Neuron Differentiation in Neuroblastoma Patients

**Figure 1:**
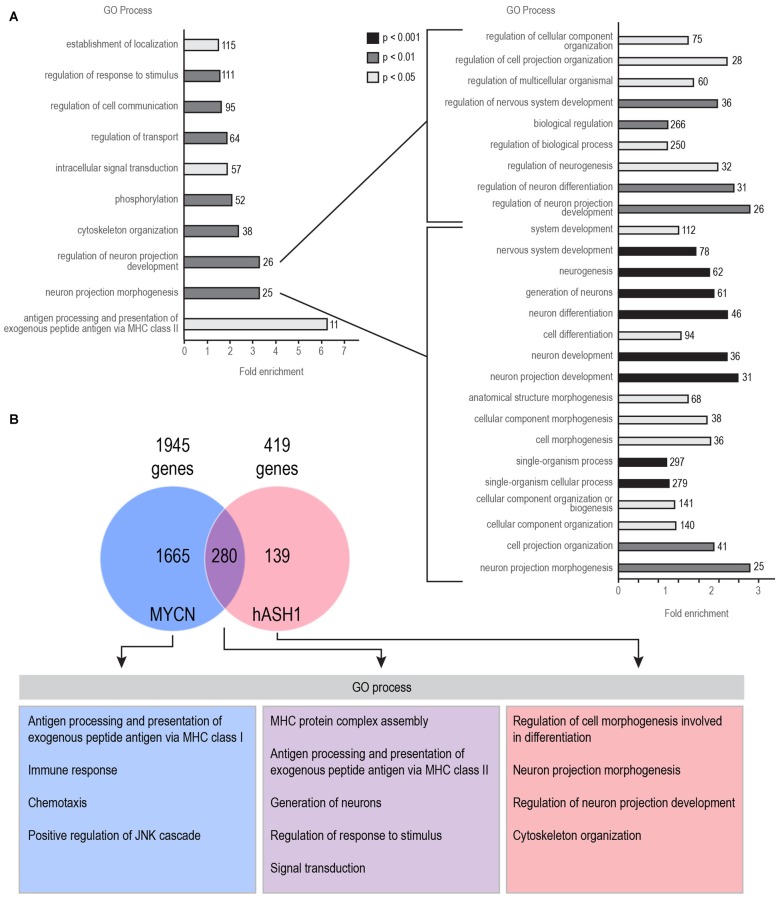
**Human achaete-scute homolog 1 (hASH1) is negatively correlated with genes associated with neurite outgrowth in neuroblastoma**. **(A)** Enrichment of Gene ontology (GO) biological processes in the 419 genes negatively correlated with hASH1 (*R* value < −0.3 and with a *p* value < 0.01) using the Kocak dataset (Kocak et al., 2013). This corresponds to the top 10% of the strongest correlated genes. A detailed view of all the categories related to regulation of neuron projection development and neuron projection morphogenesis is shown on the right. Each biological process is color coded according to its GO analysis *p* value as shown and the number of genes identified is also indicated. **(B)** Genes negatively correlated with hASH1 and N-myc (MYCN) were compared. GO analysis was performed separately for genes correlated with either MYCN or hASH1 and for the genes associated with both. A detailed list of all the GO processes and associated *p* values is provided in Supplementary Table S6. Note that the genes correlated only with hASH1 belong to the GO categories of neuron projection morphogenesis and development.

We first identified genes correlated with either hASH1 or MYCN, the latter being a well-known proto-oncogene with clearly established prognostic value in neuroblastoma patients. We analyzed three datasets that are deposited in the R2 Genomics Analysis and Visualization platform^2^: the Versteeg dataset included 88 patient samples (Valentijn et al., 2012), the Asgharzadeh dataset contained 249 patient samples (Russell et al., 2015) and the Kocak dataset contained 649 patient samples (Kocak et al., 2013) (Table 1). The overall small number of genes correlated with hASH1 relative to MYCN highlights the specificity of its putative target group (Table 1). With focus on hASH1, we further narrowed our list for the Kocak dataset to the top most correlated genes by setting a cut off for the correlation coefficient (*R* value) of ≤ −0.3 or ≥ +0.3, which reduced the number of negatively and positively correlated genes to 419 and 809, respectively. The list of identified genes correlated with hASH1 for all three datasets along with their associated *R* and *p* values is given in Supplementary Tables S2–S4. We next performed gene ontology analysis^3^ in order to determine the significantly enriched biological processes (*p* ≤ 0.05) in these gene lists. Notably, the enriched biological processes were consistent with the known functions of hASH1 in cell proliferation and differentiation (Castro et al., 2011). This is exemplarily shown in Figure 1A for the negatively correlated genes identified in the Kocak dataset, which contained the largest number of patient samples. We observed a striking association of the negatively correlated genes predominantly with processes involved in neuron projection development (e.g., regulation of neuron differentiation, regulation of neurogenesis, regulation of cell projection organization) and neuron projection morphogenesis (e.g., neuron development, neuron differentiation, generation of neurons). These findings were independently validated in the Asgharzadeh dataset (Supplementary Table S5). Despite the smaller number of negatively correlated genes in the Versteeg dataset, the only significantly enriched biological processes belonged to the category “regulation of neuron projection development”, thus further supporting a negative influence of hASH1 on neuron differentiation (Supplementary Table S5). While a myriad of biological processes were highly represented in the positively correlated genes in all three datasets, we found that they were enriched for GO biological processes corresponding to various aspects of the cell cycle (e.g., G1/S phase transition, G2/M transition of the mitotic cell cycle, mitotic interphase, spindle checkpoint), DNA replication (e.g., DNA strand elongation, DNA metabolic processes), telomere maintenance and DNA damage response and repair (e.g., double-strand break repair, cellular response to DNA damage stimulus; Supplementary Table S5). Compared to the positively correlated genes, the negatively correlated genes had fewer biological processes associated with them, and the level of significance was lower (*p* ≥ 10^−16^ vs. *p* ≥ 10^−96^ for processes associated with the positively correlated genes). However, processes involving neurogenesis and neuron differentiation were the only significant associations among the negatively correlated genes that were consistently seen across all three datasets (Supplementary Table S5). We also identified a significant association of hASH1 with neuroblastoma stage and MYCN amplification status, pointing to its functional importance in neuroblastoma (Supplementary Figures S1, S2).

**Table 1:** **Number of genes correlated with human achaete-scute homolog 1 (hASH1) and N-myc (MYCN) in human neuroblastoma**.

DATASET	Number of samples	Number of correlated genes*			
		hASH1		MYCN	
		Negative	Positive	Negative	Positive
Versteeg	88	103	366	1050	1148
Asgharzadeh	249	574	1355	3456	2405
Kocak	649	4528	4655	5896	5732

*A cutoff of p < 0.01 was used.

To exclude MYCN from mediating this effect, we compared the profile of genes correlated with both hASH1 and MYCN (Figure 1B). Despite the high overlap of genes (280/419 genes) between the two, the GO processes neuron projection morphogenesis and regulation of neuron projection development were significantly over-represented in the 139 genes not correlated with MYCN, highlighting hASH1 function in regulating these processes (Figure 1B). Interestingly, GO analysis of the MYCN correlated genes identified primarily categories associated with the immune response (e.g., T-helper 1 type immune response, regulation of T cell proliferation, T cell selection, interferon gamma mediated signaling pathway; Supplementary Table S6). These findings were corroborated in both of the other datasets (data not shown). Together, these gene expression data from three independent experiments containing a total of 986 human neuroblastoma samples reveal a significant negative correlation between hASH1 and genes involved in neuron differentiation in neuroblastoma that is independent of MYCN function.

### Characterization of RA-Induced Differentiation in Neuroblastoma Cell Lines

**Figure 2:**
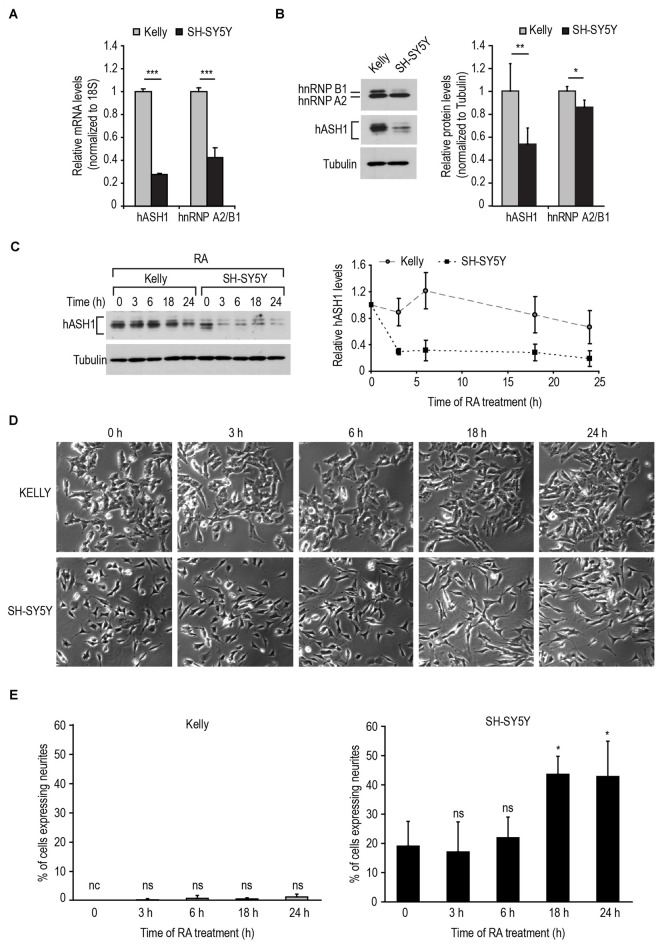
**Response of neuroblastoma cell lines to retinoic acid (RA)-induced differentiation is dependent on their hASH1 levels. (A)** hASH1 and heterogeneous nuclear ribonucleoprotein A2/B1 (hnRNP A2/B1) mRNA as determined in Kelly and SH-SY5Y cells by real-time PCR. Values were normalized to 18S ribosomal RNA and significant differences between the cell lines indicated with asterisks (***p < 0.001, *n* = 6). **(B)** Western blot analysis of hASH1 and hnRNP A2/B1 protein levels in Kelly and SH-SY5Y cells. Tubulin served as a loading control. **p < 0.01 and *p < 0.05. Error bars indicate standard deviation (*n* = 6). **(C)** Quantitation by Western blot of the time-dependent decrease in hASH1 levels in Kelly and SH-SY5Y cells upon 1 μM RA treatment. **(D)** Phase contrast images showingthe time-dependent phenotypic response to 1 μM RA. Note that RA treatment causes outgrowth of neurite-like processes in SH-SY5Y cells, but not in the Kelly cells. **(E)** Percentage of neurite containing cells were defined as cells with at least one neurite extension greater than the length of the cell body. More than 100 cells for each cell type and treatment were counted using ImageJ software, and the percentage of cells with neurite extensions are indicated. *p < 0.05, nc: not counted as no cells matching the above criteria were observed, and ns = not significant. Error bars indicate standard deviation (*n* = 3).

To reconcile these findings with the known function of hASH1 in neurogenesis, we postulated that an increase in hASH1 expression would have an inhibitory influence on neuron differentiation. Therefore, to investigate the effect of hASH1 levels on neuron differentiation, we selected two neuroblastoma cell lines derived from metastatic bone tumor biopsies, Kelly and SH-SY5Y, expressing endogenous high and low levels of hASH1, respectively. As neuroblastomas are also genetically characterized based on their genomic MYCN amplification status and given the observed correlation with hASH1 (Supplementary Figure S2), the Kelly cells with the MYCN-amplified genome and the MYCN-non-amplified SH-SY5Y cells also enabled us to dissect the contribution of hASH1 independent of MYCN amplification. Since we have previously shown that hASH1 is regulated by hnRNP A2/B1 (Kasim et al., 2014), we monitored the levels of both hASH1 and hnRNP A2/B1 in these cells. Real-time PCR analysis revealed higher levels of both hASH1 and hnRNP A2/B1 mRNA in Kelly vs. SH-SY5Y cells (Figure 2A). Western blot analysis confirmed the high levels of hASH1 protein in Kelly cells and the relatively low levels in SH-SY5Y cells (Figure 2B), the multiple protein bands likely reflecting post-translational modifications (Wylie et al., 2015). We detected overall lower hnRNP A2/B1 protein levels in SH-SY5Y than in Kelly cells, which was primarily due to the decreased levels of the hnRNP B1 isoform (Figure 2B). Both cell lines expressed neuropeptide Y and neuronal marker genes, i.e., growth associated protein 43 and microtubule-associated protein 2, confirming their neuronal character (Supplementary Figure S3).

**Figure 3:**
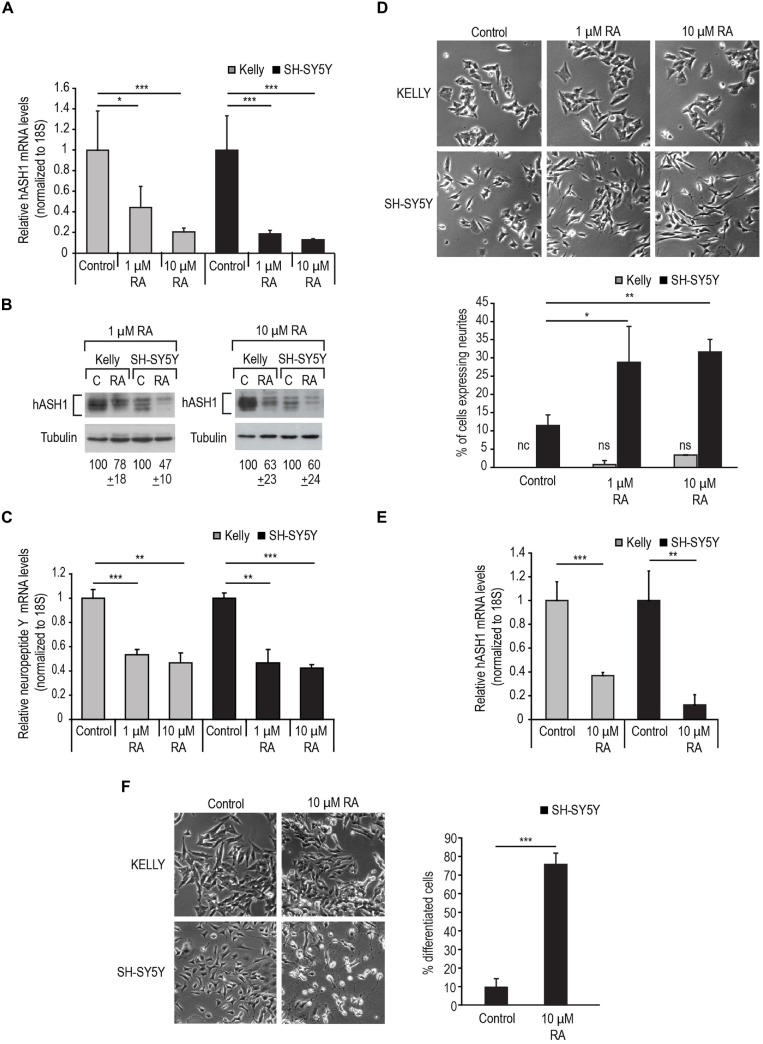
**Kelly cells are responsive to RA but resistant to RA-induced differentiation. (A)** Decrease in hASH1 levels upon 1 and 10 μM RA treatment at 24 h monitored by real-time PCR and **(B)** Western blot analysis.***p < 0.001 and *p < 0.05. Error bars indicate standard deviation (*n* = 6). **(C)** Real-time PCR analysis of NPY levels upon RA treatment as in **(A)**. Significant differences are indicated with asterisks ***p < 0.001 and **p < 0.01. Error bars indicate standard deviation (*n* = 3). **(D)** Phase contrast images showing concentration dependent effect of RA on the morphology of Kelly and SH-SY5Y cells. Note the formation of neurite-like processes in the RA-treated SH-SY5Y cells, whereas Kelly cells did not respond morphologically to RA. Percentage of neurite containing cells were counted as in Figure 2E. **p < 0.01, *p < 0.05, nc: not counted as no cells matching the above criteria were observed, and ns: not significant. Error bars indicate standard deviation (*n* = 3). **(E)** Kelly and SH-SY5Y cells were treated with 10 μM RA for 4 days and hASH1 mRNA levels monitored by real time PCR analysis. Values were normalized to 18S ribosomal RNA and significant differences indicated with asterisks (**p < 0.01 and ***p < 0.001). Error bars indicate standard deviation (*n* = 3). **(F)** Kelly and SH-SY5Y cells were treated with 10 μM RA and imaged at day 4. Percentage of differentiated cells were defined as those with neurite extensions at least two times the length of the cell body. The total number of cells were counted for each cell type and treatment and the percentage of differentiated cells for the SH-SY5Y cells indicated. ***p < 0.001. Error bars indicate standard deviation (*n* = 3). Note that for the Kelly cells, the neurite extensions did not fit our criteria to be counted as differentiated.

We then used RA to differentiate the cells toward a neuronal phenotype. We first tested the response of the two cell lines to 1 μM RA (Figure 2C). As a rapid reduction in hASH1 levels upon RA treatment of SH-SY5Y cells has been reported (Söderholm et al., 1999), we monitored the decrease in hASH1 levels by Western blot analysis in both cell lines in a time course of up to 24 h. As expected, SH-SY5Y cells displayed a rapid decrease in hASH1 levels that was noticeable as early as 3 h (Figure 2C). Surprisingly, the Kelly cells maintained stable hASH1 expression at early time points with a decline in hASH1 levels becoming evident only at 18–24 h, which did not reach the low levels observed in the SH-SY5Y cells (Figure 2C). More interestingly, phase contrast microscopy revealed that these changes in hASH1 levels correlated with the cells ability to undergo the morphological changes associated with neuronal differentiation (Figures 2D,E). Although the SH-SY5Y cells contain small neurites even in the absence of retinoids, RA treatment enhanced neurite outgrowth in these cells. However, no similar morphological change was observed in the Kelly cells. Both cell lines responded to RA by down-regulating hASH1 mRNA and protein confirming that RA was indeed active in these cells (Figures 3A,B). Consistently, both cell lines also responded to RA by decreasing neuropeptide Y levels (Figure 3C); a decrease in levels of neuropeptide Y in RA-treated SH-SY5Y has already been documented (Edsjö et al., 2004). Although a higher concentration of RA was more effective at stimulating differentiation as assessed by the development of neurite-like processes in the SH-SY5Y cells (Figure 3D), the Kelly cells remained morphologically resistant to RA and lacked the neurite-like processes despite a similar down-regulation of hASH1 mRNA and protein levels (Figures 3A–C). Longer RA treatment times up to 4 days confirmed the resistance of majority of the Kelly cells to RA-induced differentiation (Figures 3E,F). The SH-SY5Y cells, on the other hand, showed an intense neurite network after a 4 day exposure to RA and a concomitant strong down-regulation in hASH1 (Figures 3E,F). Although the variability inherent to different cell lines raises the possibility of cell-type specific effects in the regulation of RA-induced differentiation, these data suggest that high hASH1 expression during the early phase of RA treatment inhibits the phenotypic changes associated with retinoid induced differentiation.

### hASH1 Suppresses RA-Induced Differentiation

**Figure 4:**
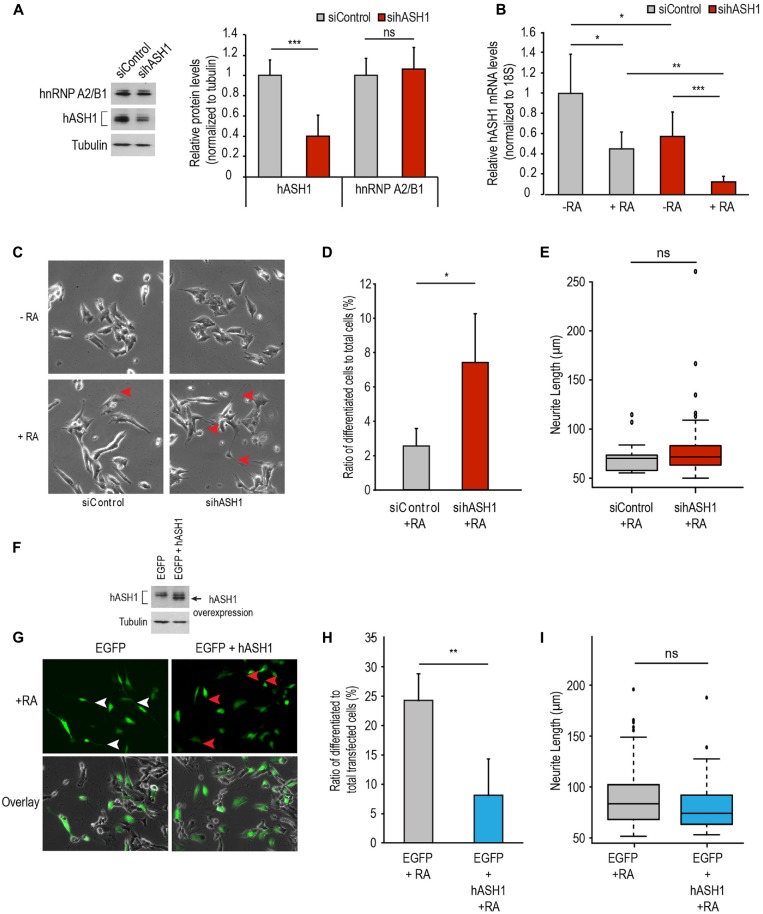
**hASH1 levels affect RA-induced differentiation in Kelly and SH-SY5Y cells. (A)** siRNA mediated knock-down of hASH1 was performed in Kelly cells and cells were harvested following 2 days of siRNA transfection (10 nM final concentration). A representative Western blot experiment is shown demonstrating knock-down efficiency at the protein level (left panel) and quantitation of the data from *n* = 9 (right panel). **(B)** Kelly cells from **(A)** were allowed to differentiate for 4 days and the decrease in hASH1 levels monitored by real time PCR. Values were normalized to 18S ribosomal RNA and significant differences indicated with asterisks (***p < 0.001, **p < −0.01 and *p < 0.05). *n* = 8. **(C)** Kelly cells were imaged at day 4 of differentiation and the neurite-like processes indicated by red arrowheads. **(D)** Cells from RA treated siControl and sihASH1 were counted and neurites measured using NeuronJ software (*p < 0.05; total number of cells counted for siControl + RA = 680 and sihASH1 + RA = 776). Data is expressed as the ratio of differentiated cells to total cells counted. **(E)** Box plot analysis of neurite length of the differentiated cells from siControl and sihASH1. ns = not significant. **(F)** Western blot analysis of total cell populations of SH-SY5Y cells over-expressing EGFP or EGFP + hASH1. Tubulin served as a loading control. **(G)** SH-SY5Y cells overexpressing EGFP or EGFP + hASH1 were enabled to differentiate with RA and imaged at day 4. Long neurite-like processes are indicated by white arrowheads in the EGFP overexpressing cells and the shorter processes with red arrowheads in the EGFP + hASH1 overexpressing cells. **(H)** RA treated transfected cells were counted and neurites measured using NeuronJ software (total number of cells counted for EGFP + RA = 437 and EGFP + hASH1 + RA = 446). Data are expressed as the ratio of differentiated cells to total transfected cells counted. **(I)** Box plot analysis of neurite length of the differentiated cells from control and hASH1 transfected cells. ns = not significant.

To determine if indeed hASH1 is responsible for the observed lack of responsiveness of the Kelly cells to RA, we silenced hASH1 in these cells by RNA interference. The efficiency of silencing was determined by Western blot analysis at 2 days post-transfection (Figure 4A). Interestingly, knock-down of hASH1 had no effect on hnRNP A2/B1 that we have shown previously to regulate hASH1 levels (Kasim et al., 2014). We next investigated the response of these Kelly cells with high (siControl) and with reduced hASH1 levels (sihASH1) to RA treatment for 4 days. We found that both siControl and sihASH1 cells responded effectively to RA by down-regulating hASH1 (Figure 4B). The sihASH1 cells retained their lower level of hASH1 for the course of the experiment (Figure 4B, compare siControl to sihASH1) and their lower hASH1 level is also reflected in their significantly lower levels of hASH1 at the end of RA treatment (Figure 4B, compare siControl + RA to sihASH1 + RA). Morphological examination by phase contrast microscopy revealed that in comparison to siControl cells, more RA-treated si-hASH1 Kelly cells developed neurite-like processes (Figures 4C,D). There was no significant difference in neurite length between differentiated cells obtained from the high and low hASH1 expressing cells (Figure 4E). Qualitatively similar results were obtained by silencing hnRNP A2/B1 that has previously been documented to decrease hASH1 protein levels (Supplementary Figure S4; Kasim et al., 2014), thus providing additional evidence for the inhibitory effect of high hASH1 on neuronal differentiation.

The data described above indicate that hASH1 prevents RA-induced differentiation of neuroblastoma cells. To further prove the specificity of hASH1 in regulating this effect, we examined the hASH1-dependent RA-differentiation response in SH-SY5Y cells that have a lower basal expression of hASH1. We transiently overexpressed hASH1 in these cells (Figure 4F). Interestingly, ectopically expressed hASH1 had a lower molecular mass indicative of the non-phosphorylated form of hASH1 and unlike endogenous hASH1 that is phosphorylated in neuroblastoma cell lines (Wylie et al., 2015). Co-transfection with EGFP allowed for visualization of hASH1 transfected cells by fluorescence microscopy and allowed for live-cell imaging to monitor cell morphology following RA treatment. Since the hASH1 and EGFP plasmids were transfected at a 9:1 ratio, fluorescent cells are expected to overexpress hASH1. We confirmed the overexpression of hASH1 in the co-transfected cells by immunofluorescence microscopy and by co-localization of hASH1 and EGFP (Supplementary Figure S5). The EGFP + hASH1 co-transfected cells expressed up to 9.6-fold higher levels of hASH1 compared to the control EGFP transfected cells (Supplementary Figure S5). These transfected cells were then allowed to differentiate with RA for 4 days. The EGFP alone expressing cells also served as a transfection control and developed long neurites by day 4 of differentiation (Figure 4G, left upper panel, white arrowheads), while hASH1 overexpression strikingly diminished neurite extension in the majority of the cells (Figure 4G, right upper panels, red arrowheads). Notably, hASH1 overexpression resulted in a significant decrease in the number of differentiated cells (Figure 4H). There was no significant difference in neurite length between the differentiated cells from the control or high hASH1 overexpressing cell line (Figure 4I). Overlay of the fluorescent images on the phase contrast images confirmed the efficacy of RA to induce differentiation in both cell populations as the non-transfected cells in each showed a similar degree of neurite outgrowth (Figure 4G, lower panels). As EGFP alone had no influence on neurite extension, we conclude that overexpression of hASH1 inhibits neurite extension. Thus, our findings in the two cell lines reveal a universal role for hASH1 in regulating RA differentiation.

### hASH1 Represses Retinoic Acid Receptor Element-Dependent Transcription

**Figure 5:**
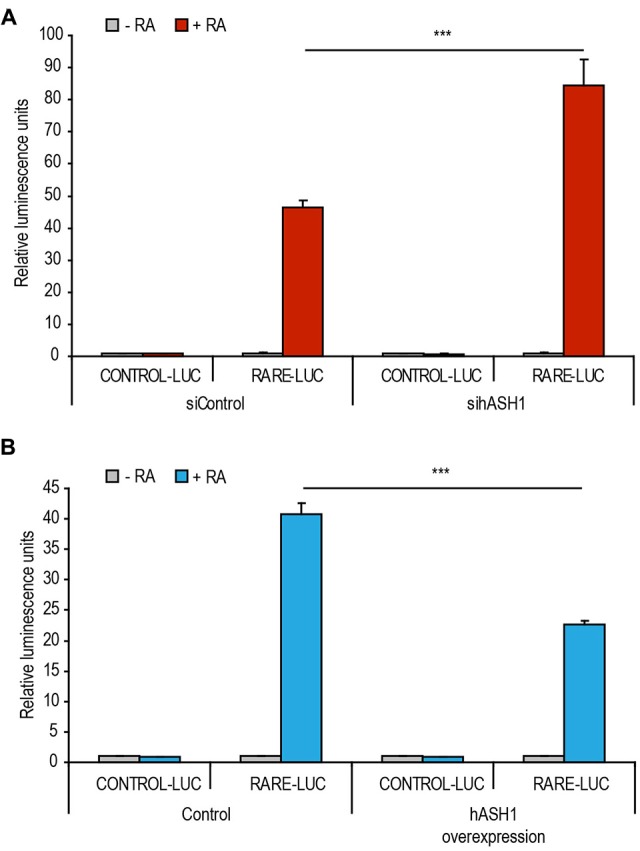
**hASH1 represses RA-dependent transcription at the retinoic acid response element (RARE). (A)** Kelly cells transfected with either control siRNA or siRNA targeting hASH1 or **(B)** SH-SY5Y cells transfected with control plasmid or a plasmid expressing hASH1 were also transfected with a control luciferase (CONTROL-LUC) reporter construct or a luciferase plasmid containing the RARE-LUC. Transcription of RARE-LUC is thus dependent on RA. Luminescence values were normalized to renilla activity. Asterisks indicate significant changes with ***p < 0.001, *n* = 6.

To gain insight into the molecular mechanism behind hASH1 repression of RA-induced differentiation, we examined the effect of hASH1 on RA-dependent transcription at a retinoic acid response element (RARE) in the context of a luciferase reporter construct. We first reduced hASH1 levels by siRNA-mediated knockdown in Kelly cells prior to RA treatment. As shown in Figure 5A, a 1.8 fold increase in RA-dependent luciferase activity upon hASH1 knockdown was observed, indicating a hASH1-dependent repression of retinoid activity. We then compared the retinoid activity upon hASH1 overexpression in SH-SY5Y cells and found that luciferase activity was significantly lower upon hASH1 overexpression, again indicating a hASH1-dependent repression of retinoid activity (Figure 5B). Collectively, these data show that hASH1 represses RA-dependent transcription at the RA receptor element.

### Hypoxic Preconditioning Promotes RA-Differentiation of High hASH1-Expressing Cells

**Figure 6:**
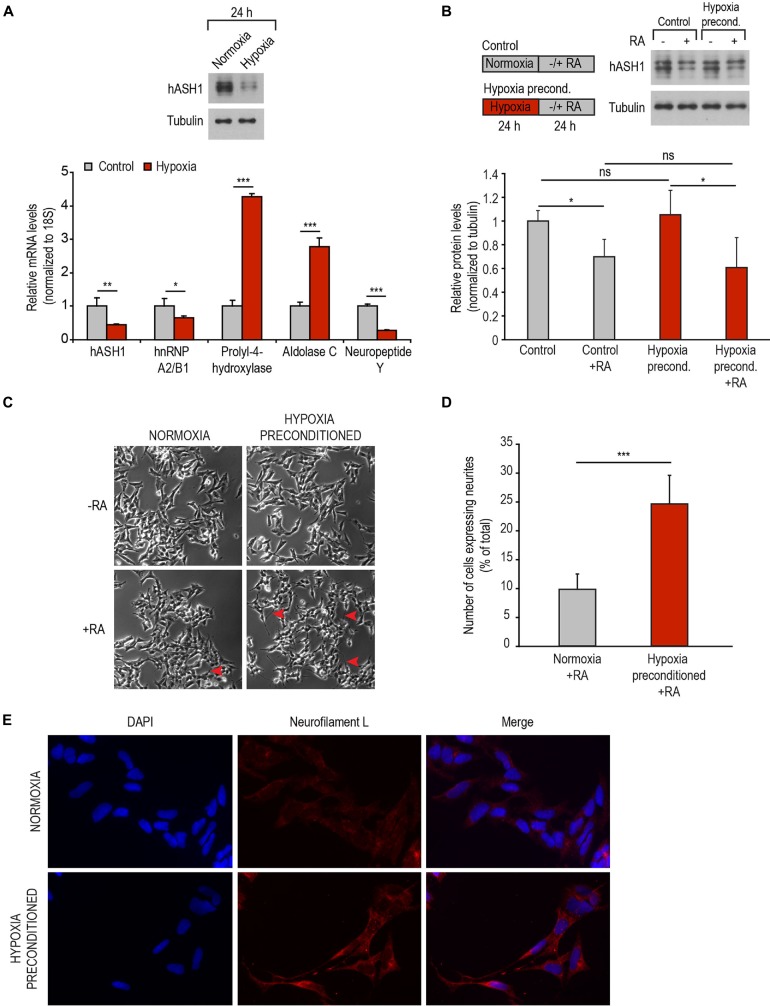
**Hypoxic preconditioning promotes RA-induced differentiation. (A)** Representative Western blot showing decreased levels of hASH1 following 24 h of hypoxia (top panel). Tubulin served as a loading control. Efficacy of hypoxia treatment was monitored by real-time PCR analysis of hypoxia marker genes prolyl-4-hydroxylase and aldolase C. hASH1, hnRNP A2/B1 and neuropeptide Y levels are shown in addition (bottom panel). Values were normalized to 18S ribosomal RNA. Significant differences are indicated with asterisks ***p < 0.001, **p < 0.01, *p < 0.05; *n* = 6. **(B)** Representative Western blot showing hASH1 levels of control cells and cells preconditioned to hypoxia following RA treatment. Schematic of the experimental design is shown in the left panel. Values were normalized to tubulin. Significant differences are indicated with an asterisk *p < 0.05 and ns: not significant; *n* = 6. **(C)** Phase contrast images of cells as described in **(B)**. Neurite-like processes in the hypoxia preconditioned cells are indicated by red arrows. **(D)** The number of RA treated cells expressing neurites and the total number of cells were counted using NeuronJ software (***p < 0.001; total number of cells counted for normoxia + RA = 2224 and hypoxia preconditioned + RA = 2139). **(E)** Immunofluorescence assays showing neurofilament L staining of normoxia and hypoxia preconditioned cells treated with RA for 24 h.

To explore the possibility of promoting differentiation of the otherwise differentiation resistant Kelly cell line under physiological circumstances, we used hypoxia as a tool to first manipulate hASH1 levels (Kasim et al., 2014). To this end, Kelly cells were preconditioned to hypoxia for 24 h, a time point at which a strong reduction in hASH1 levels was observed (Figure 6A). The efficacy of hypoxia treatment was confirmed by up-regulation of hypoxia marker genes (aldolase C and prolyl-4-hydroxylase) as well as the down-regulation in NPY that has been previously documented (Figure 6A) (Jögi et al., 2002). Immediately after 24 h of hypoxic preconditioning, when these cells have lowered levels of hASH1, cells were induced to differentiate with RA under normoxic conditions. Control cells grown at normoxia for 24 h were processed similarly. Western blot analysis confirmed the down-regulation in hASH1 protein levels indicative of effective RA treatment after both normoxia and hypoxia growth conditions (Figure 6B). It is worth mentioning that no significant difference was observed in hASH1 levels between the control and hypoxia preconditioned cells when placed in normoxia for 24 h (Figure 6B). However, only the hypoxia preconditioned cells, with lower hASH1 levels at the time of RA treatment, displayed retinoid-induced neurite-like processes that stained positive for neurofilament L, which is expressed during neuronal differentiation (Figures 6C–E). Taken together, our data show that manipulation of hASH1 expression by hypoxia influences the sensitivity of neuroblastoma cells to RA-induced differentiation.

## Discussion

Neuroblastoma is one of the few malignancies capable of spontaneous differentiation and regression (Maris et al., 2007). Increasing the ability to induce neuroblastoma differentiation is thus key to disease therapeutics; however, this is often impeded by the acquired refractiveness of neuroblastoma to the differentiation agent. Our data here revealed hASH1 to play a decisive role in inhibiting the differentiation of neuroblastoma in response to RA, a finding that may have important implications in differentiation therapy.

We discovered a common function of hASH1 in inducing genes involved in cell cycle and DNA repair while repressing genes involved in neuron differentiation in neuroblastoma. This was evident across a large number of neuroblastoma tumors from three different datasets that were obtained using different microarray platforms, thus supporting the clinical significance of our findings. Interestingly, this function of hASH1 in neuroblastoma mirrors that of MYCN (Valentijn et al., 2012). The ability of MYCN to repress neuron differentiation was inferred from the neuronal expression of its negatively regulated genes, most of which were not identified to be correlated with hASH1 (data not shown; Valentijn et al., 2012). It is possible that MYCN cooperates with hASH1 to exert a concerted effect, which is reflected in the observed correlation of hASH1 expression with MYCN amplification. Nevertheless, the over-representation of MYCN non-amplified tumors in the neuroblastoma datasets that we used and the regulation of the MYCN non-amplified SH-SY5Y cell differentiation by hASH1 indicates that hASH1 function in neuron differentiation in neuroblastoma is likely to be independent of MYCN amplification status. The importance of this being underlined by the fact that MYCN amplification, although the most well-known prognostic marker, is observed in only 22% of primary tumors (Brodeur and Bagatell, 2014).

Given the lack of responsiveness of neuroblastomas to RA, the negative association of hASH1 with neuron differentiation was of particular interest as it provided the first clue to the inhibitory role of hASH1 on differentiation. The two neuroblastoma cell lines we used to confirm this are ideal examples of neuroblastoma that are either responsive or non-responsive to differentiation by RA. Interestingly, it was the low hASH1 expressing SH-SY5Y cells that were capable of undergoing the morphological changes associated with differentiation. In these cells, hASH1 levels decreased more or less concomitantly with RA treatment, evident as early as after 3 h. In contrast, majority of the high hASH1 expressing Kelly cells failed to express neurite-like processes even upon prolonged RA treatment. Here, it is important to emphasize that RA was indeed active in the Kelly cells, as evidenced by the down-regulation of NPY and also hASH1; yet, despite the decreased hASH1 levels, the Kelly cells failed to differentiate in response to RA. We assume that an initial threshold level for hASH1 expression exists beyond which the inhibitory effects on differentiation are observed. Therefore, it is the basal hASH1 level at the time of RA exposure (early phase) that is crucial in determining the differentiation fate of the cells. This is particularly true for Kelly cells that do not show a rapid decrease in hASH1 protein levels upon retinoid treatment. The hASH1 levels in both cell lines correlated with hnRNP B1 that is known to have a stabilizing effect on hASH1 mRNA and protein and could also play a role in their different basal hASH1 expression and their response to RA (Kasim et al., 2014).

Several lines of evidence strongly suggest hASH1 to be involved in modulating the cellular differentiation in response to RA. First, down-regulation of hASH1 by directly targeting the hASH1 gene by RNA interference in high-hASH1 expressing Kelly neuroblastoma cells promoted RA-induced differentiation. Conversely, over-expression of hASH1 in low-hASH1 expressing SH-SY5Y neuroblastoma cells conferred resistance to RA-induced differentiation. Second, down-regulation of hASH1 by targeting a modulator of hASH1, namely hnRNP A2/B1, also promoted RA-induced differentiation. Third, hypoxic preconditioning that significantly decreases hASH1 levels prior to RA treatment also enhanced differentiation. Finally, the ability of hASH1 to modulate RA-induced differentiation correlated with its influence on transcription at the RA receptor element. The mechanistic basis behind hASH1-dependent transcription repression is not known. To date, a myriad of transcription regulatory proteins have been described to influence retinoid signaling (Gudas and Wagner, 2011; Cunningham and Duester, 2015). For instance, Zinc Finger Protein 423 was identified by a large-scale RNA interference screen to be critically required for RA-induced differentiation of neuroblastoma cells (Huang et al., 2009). Here, we identified hASH1 to inhibit RA-induced transcription at the RA receptor element, thus, acting as a possible co-repressor of retinoid signaling. In this regard, binding of several proneural proteins including Neurogenin1, Neurogenin2 and hASH1 to RA receptor in the absence of ligand has already been described (Lee et al., 2009). We cannot, however, exclude the involvement of other factors that are targets of hASH1 in mediating this response. The known function of hASH1, a proneural transcription factor belonging to the achaete-scute gene family, is to promote neurogenesis by regulating the expression of differentiation genes; a function that is conserved in all organisms (Huang et al., 2014). In *Drosophila*, achaete-scute expression confers neural identity to the developing neuroblast, whereas in mammalian systems, expression is restricted to cells that already have neural identity (Lo et al., 1991; Skeath and Carroll, 1992). Regulation of hASH1 expression is critical to the development program (Imayoshi et al., 2013). Our data suggest that aberrant regulation of hASH1, leading to high hASH1 expression, would inhibit differentiation and maintain neuronal cells in a proliferative state. It is important to keep in mind that retinoid activity *in vivo* reciprocally maintains hASH1 at carefully regulated levels that in turn determines neuronal identities (Jacob et al., 2013). Thus, the dysregulation of hASH1 in neuroblastoma could in turn be linked to defects in the RA synthesis and metabolism pathway as has been described in glioblastoma (Campos et al., 2015).

**Figure 7:**
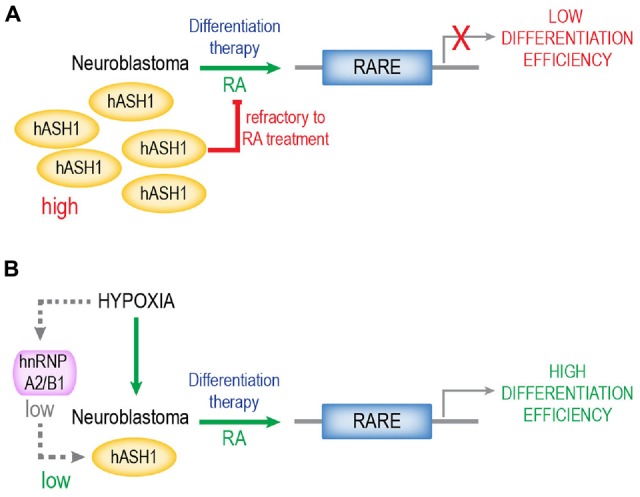
**Proposed model of hASH1 function in neuroblastoma**. High hASH1 levels in neuroblastoma inhibit RA-mediated transcription at the RARE resulting in low differentiation efficiency **(A)**. Exposure to hypoxia causes an hnRNP A2/B1-dependent decrease in hASH1 levels (Kasim et al., 2014) and thus promotes RA-induced differentiation and neurite outgrowth, resulting in higher differentiation efficiency **(B)**. Thus, hypoxic preconditioning could serve to enhance retinoid efficacy during differentiation therapy, especially for tumors refractive to RA. We propose that hASH1 levels in neuroblastoma is indicative of their differentiation potential and thus their response to differentiation therapy.

A summary of our findings is depicted in Figure 7. RA remains one of the most potent inducers of differentiation for neuroblastomas and several derivatives are used as therapy for high-risk neuroblastomas (Reynolds et al., 2003). A high tumor differentiation grade is associated with a positive outcome. Since efficacy of RA *in vivo* is limited by the refractiveness of many neuroblastomas, combination therapy with, for instance, histone deacetylase inhibitors has been applied (Hahn et al., 2008; Brodeur and Bagatell, 2014). We propose that refractiveness to RA is a function of the hASH1 levels in these tumors. One of the surprising observations that we describe is that hypoxic preconditioning remarkably enhanced RA-differentiation efficacy. These data suggest that the local oxygen environment can serve as a tool to manipulate hASH1 levels in neuroblastomas that could effectively facilitate differentiation therapy. Here, we would like to add a cautionary note and stress the point that these data were obtained from cell lines *in vitro*. The Kelly and SH-SY5Y are two different cell lines, and additional cell type-specific effects of hASH1 on the regulation of RA-induced differentiation cannot be ruled out. Despite the negative correlation between hASH1 and neuron differentiation observed in neuroblastoma patients, future *in vivo* studies aimed at evaluating the potential prognostic usefulness of hASH1 in predicting the responsiveness of these tumors to RA-based therapies are needed. Although other factors have been described to modulate RA differentiation in neuroblastoma, hASH1 represents a unique target and a possible biomarker for all neuroblastoma, independent of MYCN amplification status (Shah et al., 2014; Cimmino et al., 2015; Heynen et al., 2016). Future studies will focus on defining the mechanism of action of hASH1 during the early phase of RA-differentiation, which may reveal novel cellular targets of importance to retinoid therapy. Taken together, this study highlights hASH1 to be a modulator of retinoid-induced differentiation that is of potential clinical significance to neuroblastoma.

## Author Contributions

MK, HS and MF designed experiments; MK, VH and MF performed experiments; MK, VH, HS, PBP and MF analyzed and interpreted the data, contributed to discussion; MK and MF wrote the manuscript.

## Funding

This work was supported by the Deutsche Forschungsgemeinschaft (Grant FA 845/2-2 to MF).

## Conflict of Interest Statement

The authors declare that the research was conducted in the absence of any commercial or financial relationships that could be construed as a potential conflict of interest.

## Footnotes

^1^http://target.nci.nih.gov/dataMatrix/TARGET_DataMatrix.html

^2^http://hgserver1.amc.nl/cgi-bin/r2/main.cgi

^3^http://geneontology.org/
