# Supplementary material for: Achaete-Scute Homolog 1 Expression Controls Cellular Differentiation of Neuroblastoma
Source: Front Mol Neurosci. 2016 Dec 21;9:156. doi: 10.3389/fnmol.2016.00156 (PMC5174122; doi:10.3389/fnmol.2016.00156)
Supplement: Supplementary file 1 [file DataSheet_1.zip › New folder (2)/Kasim Supplementary Data.docx]

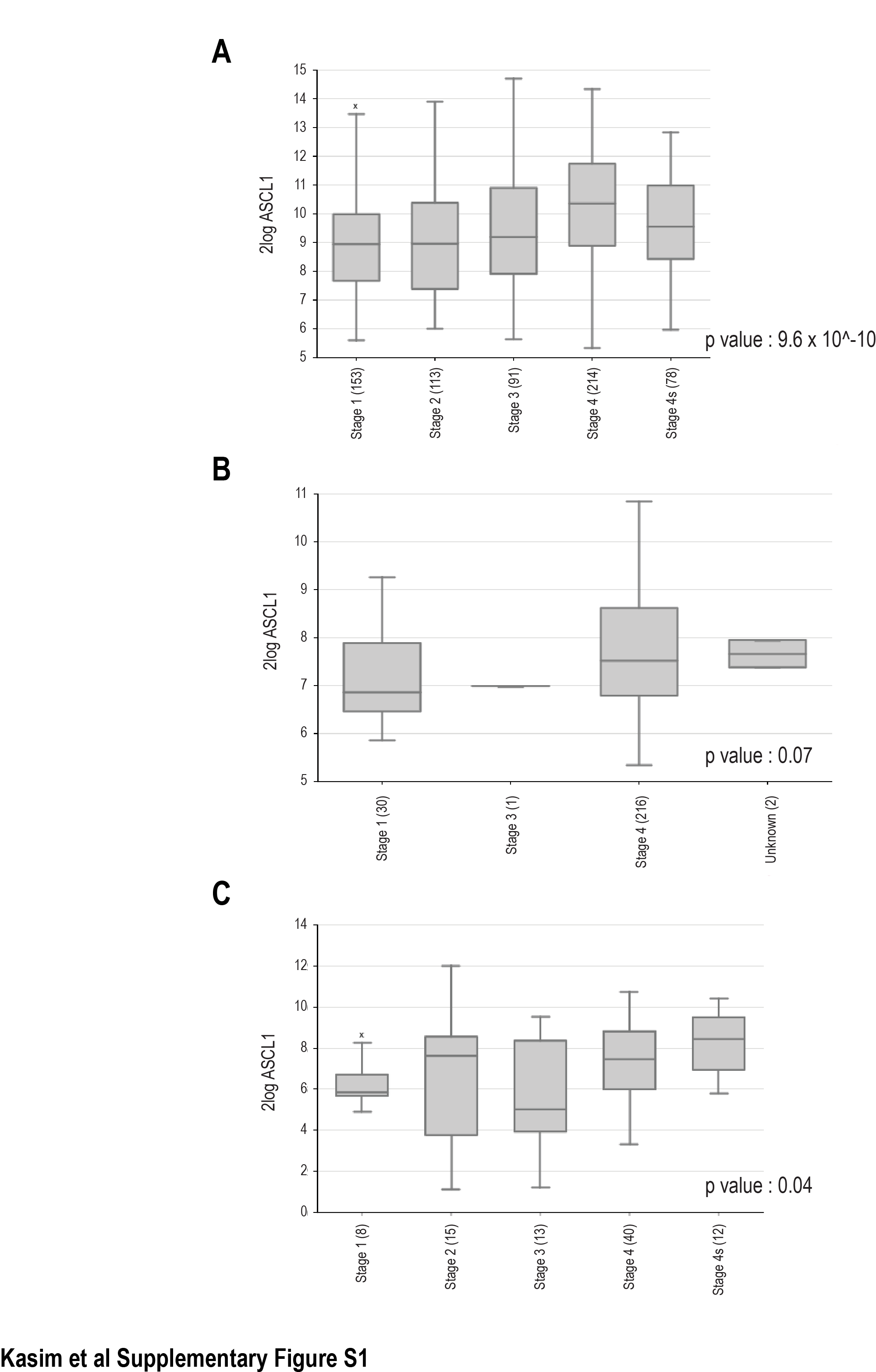


**Supplementary Figure S1. hASH1 expression is associated with disease stage in neuroblastoma.**

Box plot for hASH1 expression (2log ASCL1) per group defined by disease stage using the R2 web tool. Unknown includes samples whose disease status was not defined. The datasets used are: the Kocak dataset (n = 549) (**A)**, the Asgharzadeh dataset (n=249) **(B)** and the Versteeg dataset (n = 88) **(C)**. A one way ANOVA was performed and the p values are indicated.


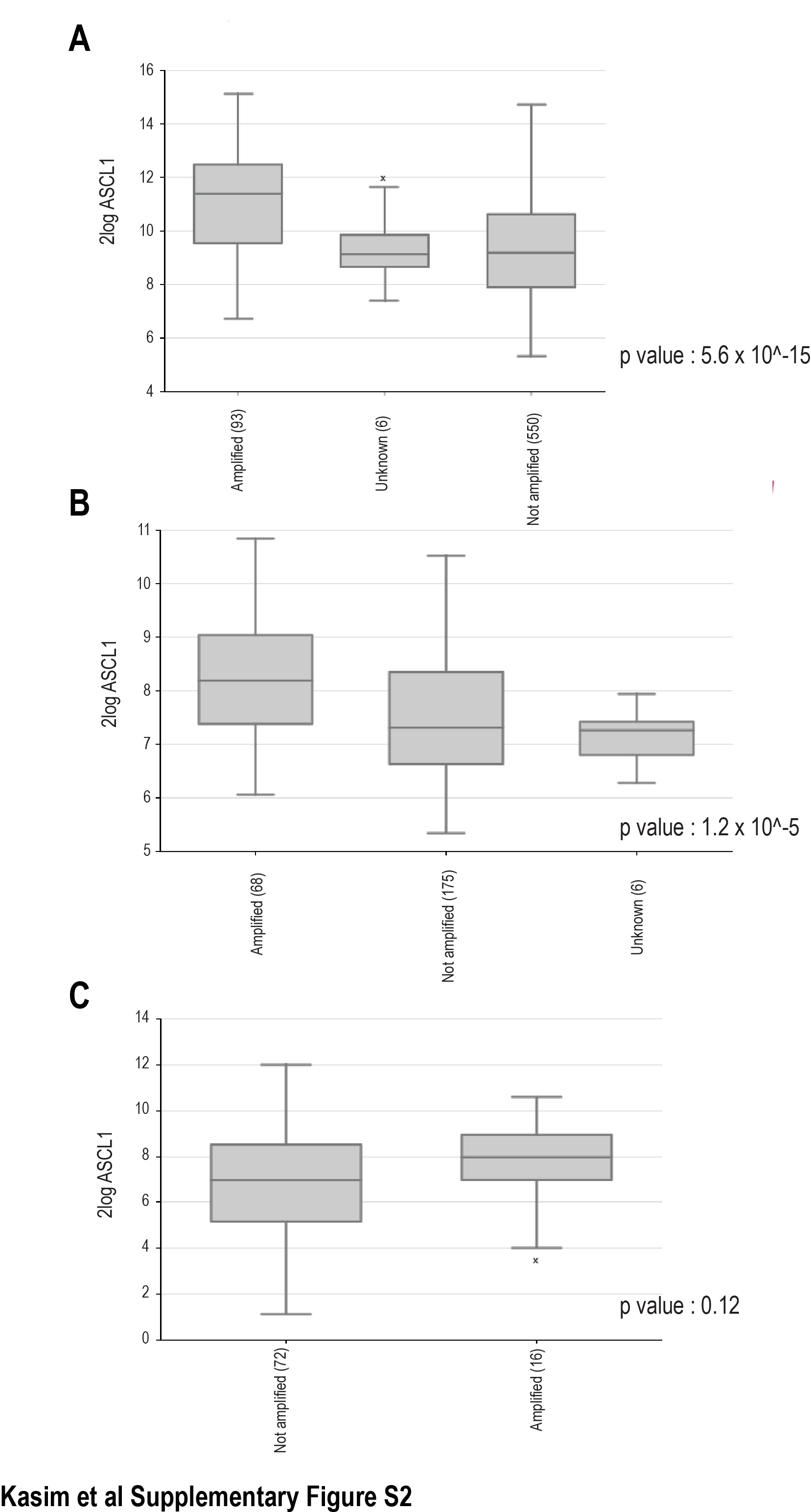


**Supplementary Figure S2**. **hASH1 expression is associated with MYCN amplification status in neuroblastoma.**

Box plot for hASH1 expression (2log ASCL1) per group defined by MYCN amplification status using the R2 web tool. Unknown includes samples whose amplification status was not defined. The datasets used are: the Kocak dataset (n = 549) **(A)**, the Asgharzadeh dataset (n=249) **(B)** and the Versteeg dataset (n = 88) **(C**). A one way ANOVA was performed and the p values are indicated.


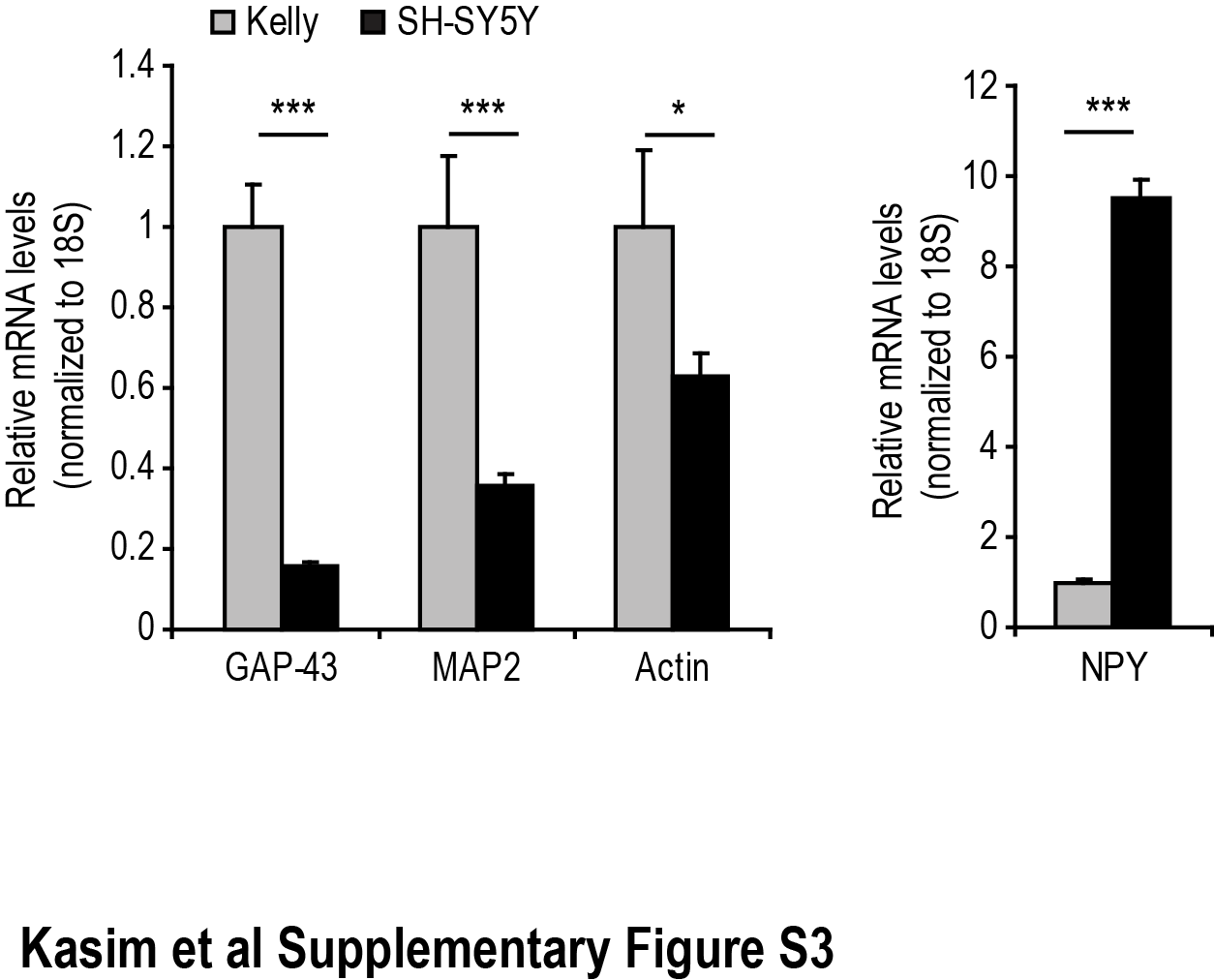


**Supplementary Figure S3**. **Expression of neuronal marker genes in Kelly and SH-SY5Y cells.**

Real-time PCR analysis of neuronal marker genes: growth associated protein 43 (GAP-43), microtubule associated protein 2 (MAP2), and neuropeptide Y (NPY), in Kelly and SH-SY5Y cells. Shown also are levels of β-actin. Values were normalized to 18S ribosomal RNA. Significant differences between the cell lines are indicated with asterisks (***: p < 0.001 and *: p < 0.05). Error bars indicate standard deviation (n = 3).


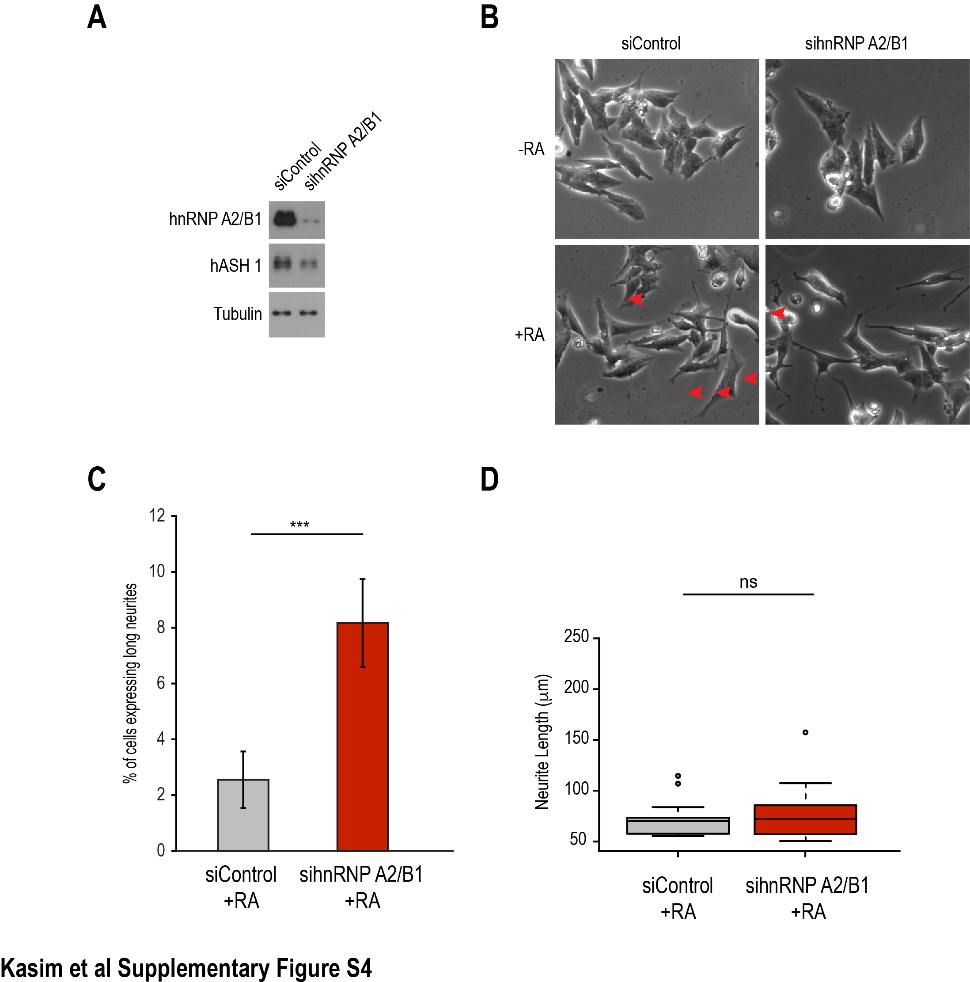


**Supplementary Figure S4**. **hnRNP A2/B1 regulates RA-induced differentiation**

**(A)** siRNA mediated knock-down of hnRNP-A2/B1 was performed in Kelly cells and cells were harvested following 48 h of siRNA transfection. Two independent experiments were performed, each in triplicate. Representative Western blot results showing knock-down efficiency of hnRNP A2/B1 and the resulting hASH1 protein levels. Tubulin served as a loading control **(B)** Phase contrast images of cells in A following 4 day treatment with 10 μM RA. Neurite-like processes indicated by red arrowheads. **(C)** RA treated cells from siControl and sihnRNP A2/B1 were counted and neurites measured using Neuron J software (***: p < 0.001; siControl+RA = 680 and sihASH1+RA = 773). Data is expressed as the ratio of differentiated cells to total cells counted. **(D)** Box plot analysis of neurite length of the differentiated cells from siControl and sihnRNP A2/B1. ns= not significant.


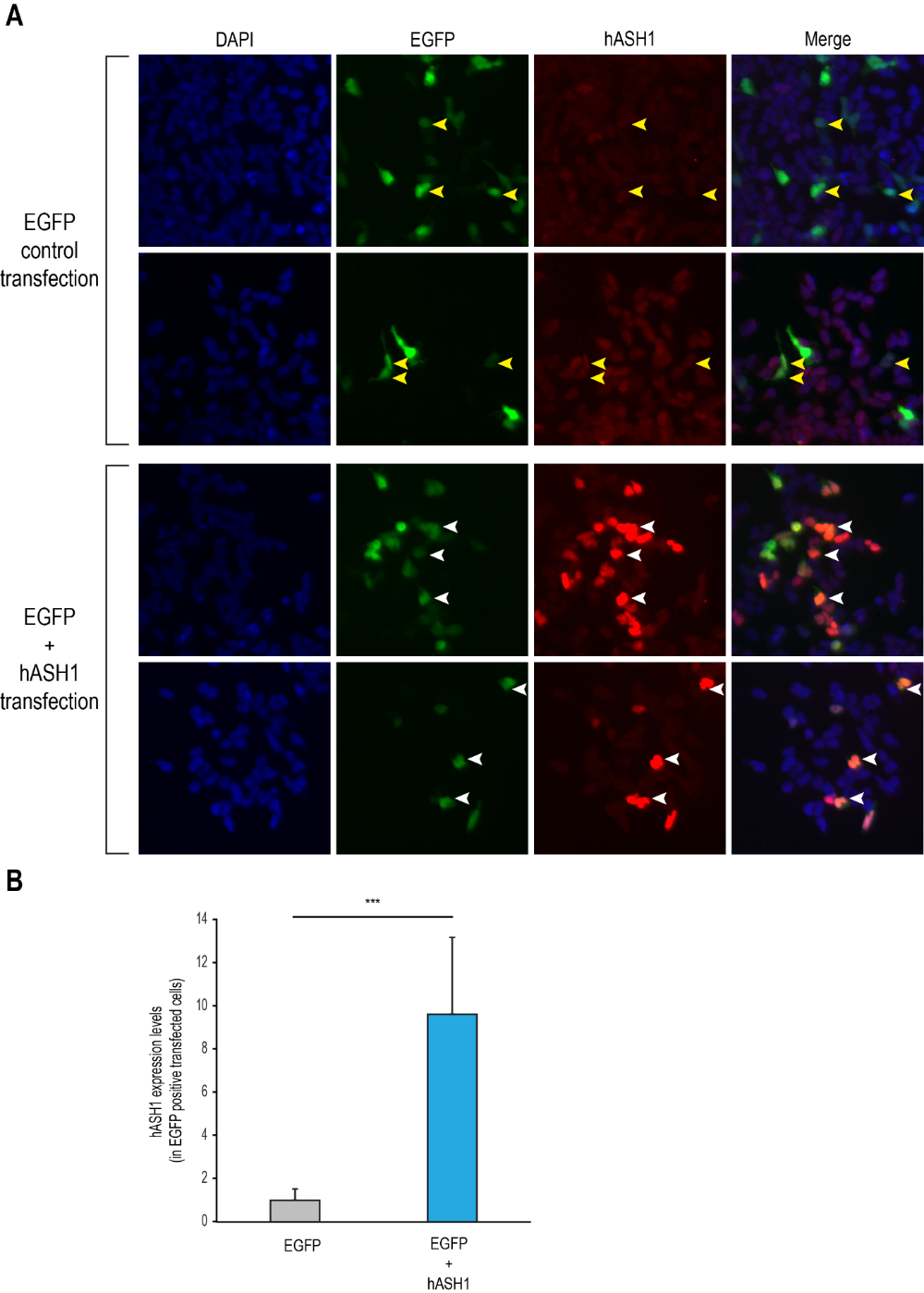


**Supplementary Figure S5**. **hASH1 is transiently overexpressed in SH-SY5Y cells.**

**(A)** Overexpression of hASH1 (red) in EGFP+hASH1 transfected SH-SY5Y cells was detected by immunofluorescence microscopy. Co-localization of the overexpressed hASH1 (red) with EGFP (green) confirmed the high expression of hASH1 in transfected cells, indicated by the white arrows in the merged panels. No overexpression of hASH1 was observed in the control transfection (yellow arrows). Nuclei were stained with DAPI (blue). Two independent experiments were performed, each in triplicate. **(B)** Quantitation of hASH1 fluorescence intensity levels in EGFP positive cells. Data were normalized to the EGFP control transfection. More than one hundred transfected cells for each treatment were counted and the hASH1 fluorescence intensity levels measured using Image J software. ***: p < 0.001.
